# Supplementary material for: Soluble Urokinase Plasminogen Activator Receptor Levels Are Associated with Severity of Fibrosis in Patients with Primary Sclerosing Cholangitis
Source: J Clin Med. 2022 Apr 28;11(9):2479. doi: 10.3390/jcm11092479 (PMC9105770; doi:10.3390/jcm11092479)

Supplementary Material:

Supplementary Table S1. Laboratory parameters of the PSC cohort.

Baseline Laboratory parameters at time of suPAR analysis (Median; Max-Min)	PSC cohort<br>n=84
**Bilirubin [mg/dL]**	0.86 (0.2-26)
**ALT [U/L]**	65 (11-429)
**AST [U/L]**	61 (12-272)
**ALP [U/L]**	191 (31-1565)
**GGT [U/L]**	126 (12-1748)
**Albumin [g/dL]**	42 (23-51)
**Creatinin [mg/dL]**	0.8 (0.3-1.7)
**CRP [mg/dL]**	4.4 (0.5-335)
**INR**	1 (0.9-2.14)
**Fibrosis--4 Index**	1.3 (0.24-22.3)
**Mayo Risk Score**	-0.2 (-1.8-3.4)
**Liver stiffness [kPA]**	8.6 (1-75)

Data are n (%) of patients, if not indicated otherwise. The percentages were rounded and may not sum 100%. Significant results (p<0.05) are shown in bold type. alanine transaminase (ALT); aspartate aminotransferase (AST); alkaline phosphatase (ALP); gamma-glutamyl transferase (GGT); international normalized ratio (INR); C-reactive protein (CRP). **Laboratory reference values**: Bilirubin < 1.2 mg/dl, ALT<31 U/l, AST 35 U/l, ALP 35-105 U/l, GGT 5-36 U/l, albumin 35-52mg/dl, creatinine 0.5-0.96 mg/dl, CRP < 5mg/dl, INR 0.9-1.25

Supplementary Table S2. Laboratory parameters of the IBD cohort.

Baseline Laboratory parameters at time of suPAR analysis (Median; Max-Min)	All IBD patients<br>n=68	Crohn's Disease<br>n=39	Ulcerative colitis<br>n=29	p-value
**Bilirubin [mg/dL]**	0.4 (0.2-1.6)	0.3 (0.2-1)	0.4 (0.2-1.6)	0.327
**ALT [U/L]**	23 (9-93)	20(11-77)	25 (9-93)	0.234
**AST [U/L]**	23 (12-68)	22 (12-55)	25 (12-68)	0.181
**ALP [U/L]**	69 (34-163)	67 (34-135)	71 (44-163)	0.624
**GGT [U/L]**	24 (8-378)	24 (11-378)	24 (8-175)	0.892
**Creatinin [mg/dL]**	0.8 (0.4-1.7)	0.8 (0.4-1.7)	0.8 (0.6-1.3)	0.327
**CRP [mg/dL]**	1.7 (0,5-58)	2 (0.5-58)	1.6 (0.5-19)	0.9
**INR**	1 (0.9-1.1)	1 (1-1.1)	1 (0.9-1.1)	0.364
**Calprotectin [µg/g]**	105 (45-800)	104 (45-800)	145 (45-800)	0.665

Data are n (%) of patients, if not indicated otherwise. The percentages were rounded and may not sum 100%. Significant results (p<0.05) are shown in bold type. Inflammatory bowel disease (IBD); alanine transaminase (ALT); aspartate aminotransferase (AST); alkaline phosphatase (ALP); gamma-glutamyl transferase (GGT); international normalized ratio (INR); C-reactive protein (CRP). **Laboratory reference values**: Bilirubin < 1.2 mg/dl, ALT<31 U/l, AST 35 U/l, ALP 35-105 U/l, GGT 5-36 U/l, creatinine 0.5-0.96 mg/dl, INR 0.9-1.25, CRP < 50mg/dl Calprotectin < 50 µg/g

Supplementary Figure S1. Circulating soluble urokinase-type plasminogen activator receptor (suPAR) levels were independent of age (**A**) Body Mass Index (BMI) (**B**), the presence of arterial hypertension (**C**), diabetes (**D**), smoking (**E**) and gender (**F**). Box plots are displayed, where the bold line indicates the median per group and the edges of the box are the first and third quartiles. The horizontal lines show minimum and maximum values of calculated nonoutlier values. The dots represent calculated outliners. Soluble urokinase-type plasminogen activator receptor (suPAR). Body Mass Index (BMI).

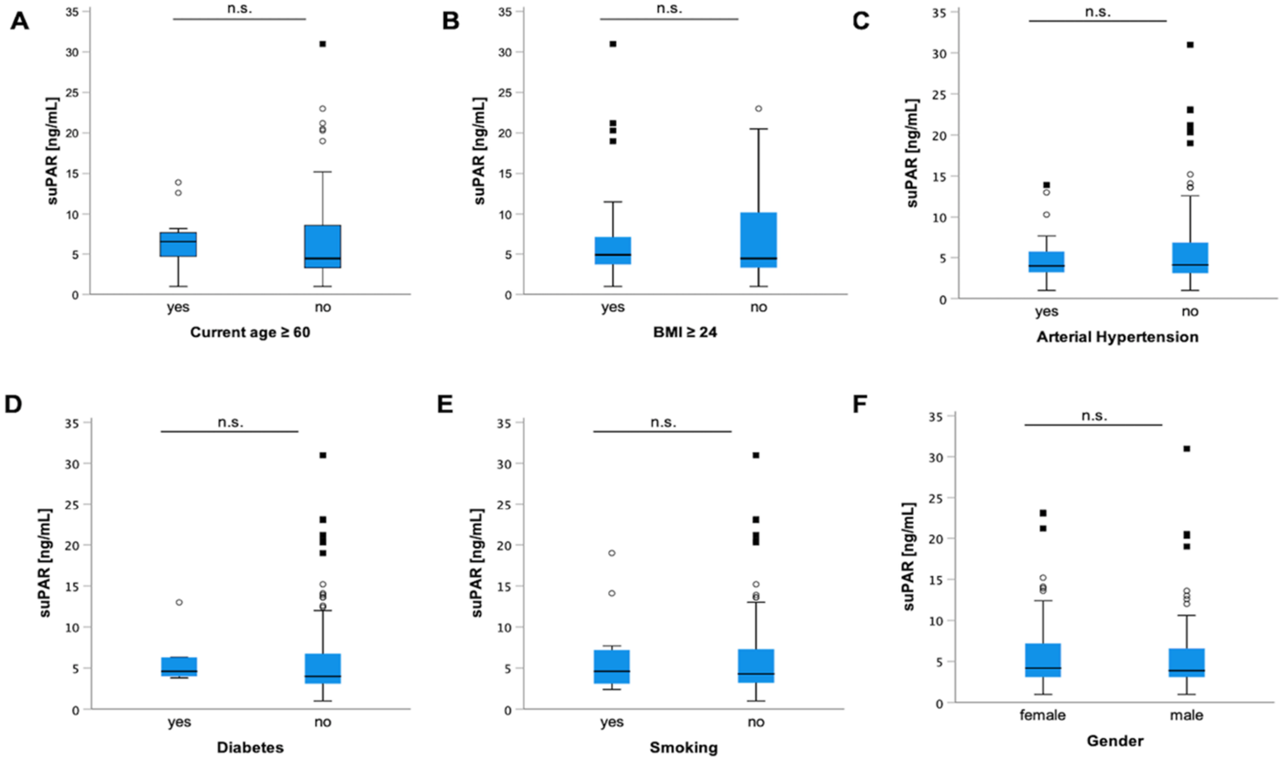

Supplementary Figure S2. Spearman rank correlation revealed a link between soluble urokinase-type plasminogen activator receptor (suPAR) and Fibrosis4 (FIB4) index ($p<0.001$; $r=0.576$) (**A**) and liver stiffness ($p<0.001$; $r=0.581$) (**B**) respectively. In line, liver synthesis parameters such as bilirubin ($p<0.001$; $r=0.525$) (**C**), albumin ($p<0.001$; $r=-0.638$) (**D**), platelet count ($p<0.001$; $r=-0.413$) (**E**) and prothrombin ($p<0.001$; $r= -0.5$) (**F**) correlated with suPAR levels. The dots represent calculated outliners. Soluble urokinase-type plasminogen activator receptor (suPAR).

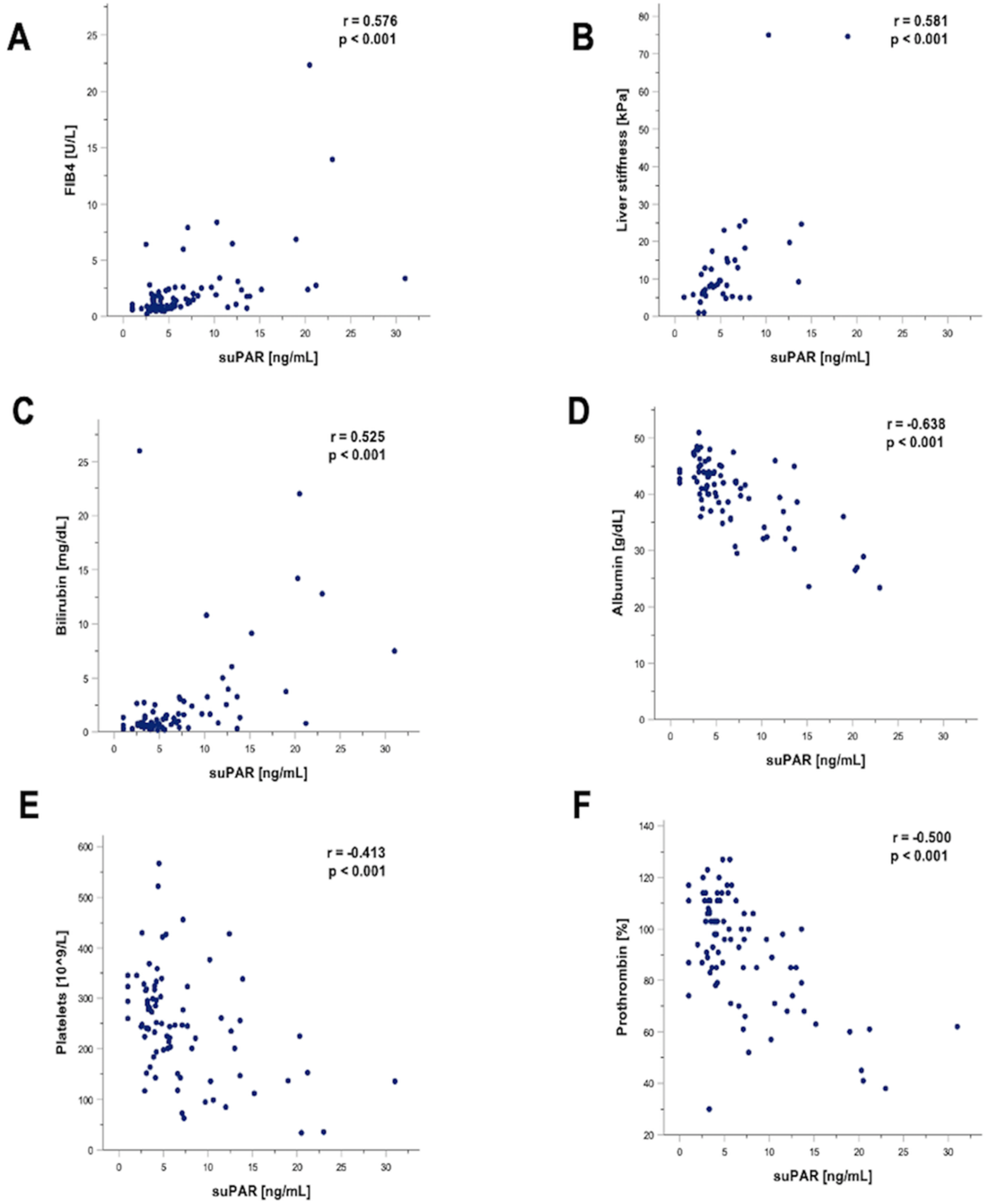

Supplementary Figure S3. Soluble urokinase-type plasminogen activator receptor (suPAR) levels are increased in patients with signs of portal hypertension. Patients with signs of portal hypertension such as ascites, esophageal varices or splenomegaly revealed higher suPAR levels compared to patients without signs of portal hypertension (p<0.001) (**A**). In particular, suPAR levels were elevated in patients with ascites (p<0.001) (**B**), esophageal varices (p<0.001) (**C**) and splenomegaly (p=0.03) (**D**). Box plots are displayed, where the bold line indicates the median per group and the edges of the box are the first and third quartiles. The horizontal lines show minimum and maximum values of calculated nonoutlier values. The dots represent calculated outliners. Soluble urokinase-type plasminogen activator receptor (suPAR). (* $p < 0.05$; *** $p < 0.001$).

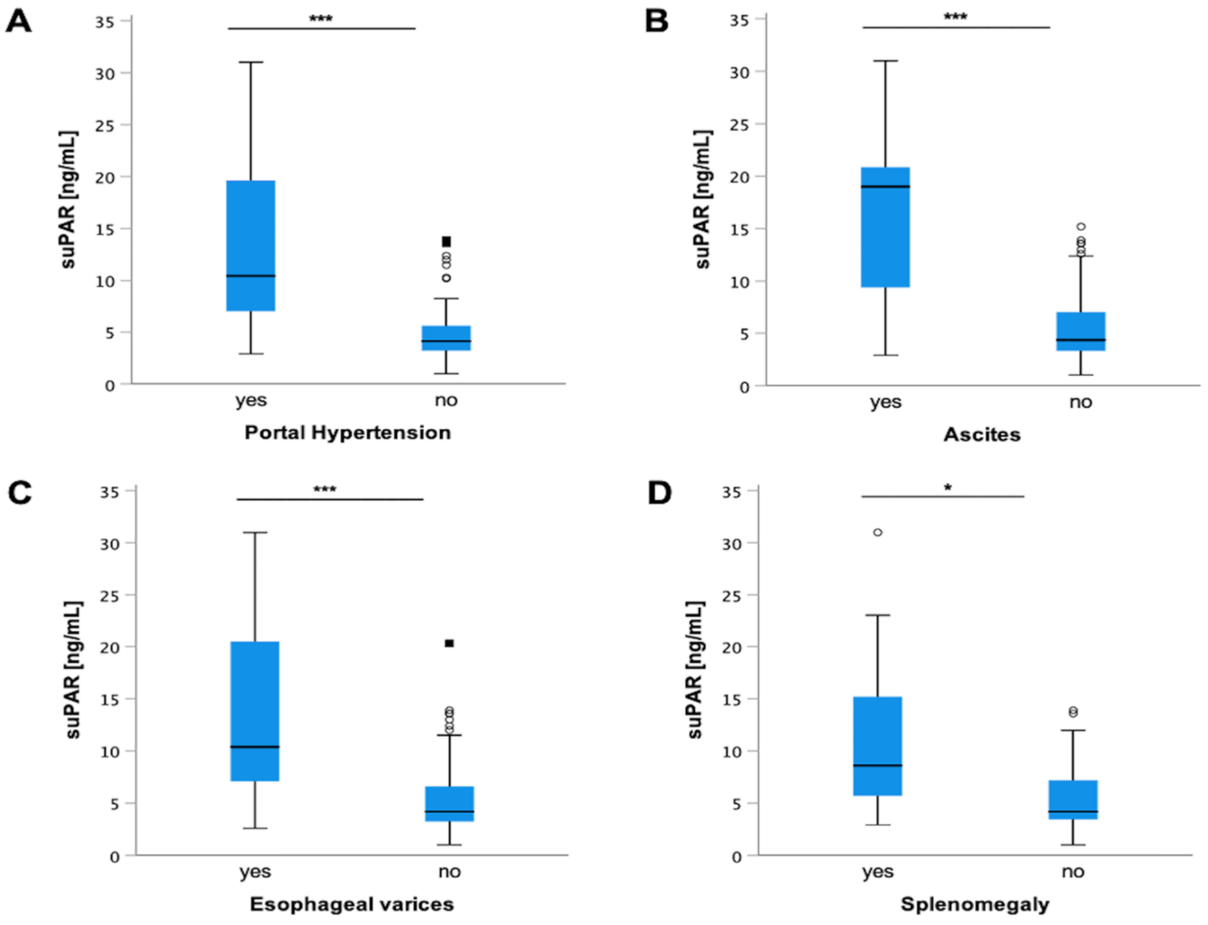

Supplementary Figure S4. Soluble urokinase-type plasminogen activator receptor (suPAR) levels are linked to prognostic parameters in PSC patients. Spearman rank correlation revealed an association of suPAR levels with prognostic markers such as the Mayo Risk score (p<0.001, r=0.649) (**A**) and alkaline phosphatase (ALP) (p=0.001; r=0.343) (**B**).

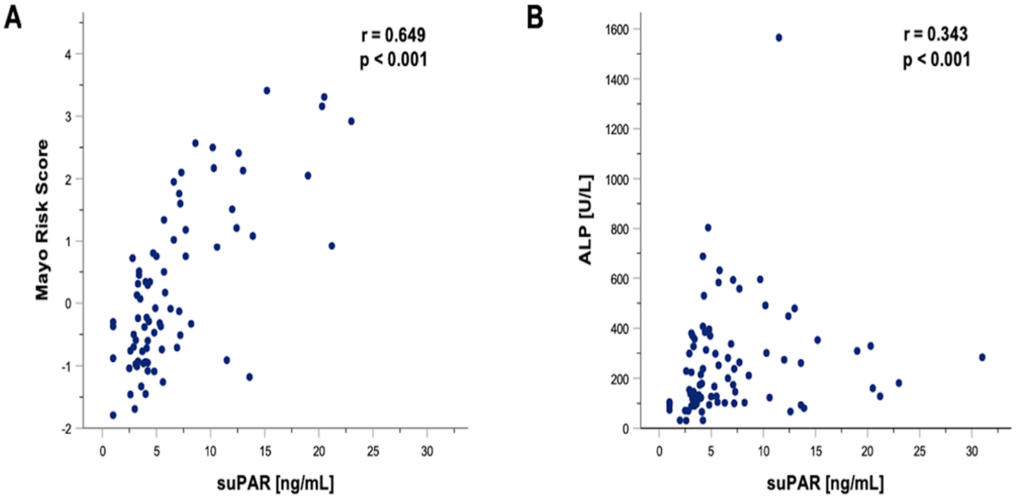

Supplementary Figure S5. Soluble urokinase-type plasminogen activator receptor (suPAR) levels in patients with PSC are independent of the presence of autoimmune hepatitis (AIH) (**A**) and inflammatory bowel disease (IBD) (**B**). Box plots are displayed, where the bold line indicates the median per group and the edges of the box are the first and third quartiles. The horizontal lines show minimum and maximum values of calculated nonoutlier values. The dots represent calculated outliners. Soluble urokinase-type plasminogen activator receptor (suPAR). Primary Sclerosing Cholangitis (PSC). Autoimmune hepatitis (AIH). Inflammatory bowel disease (IBD).

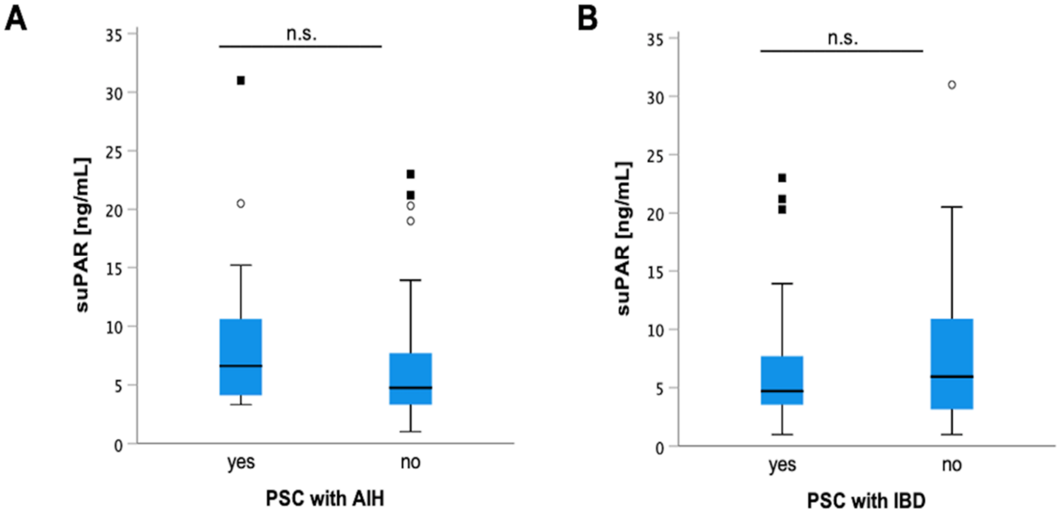

Supplement: Supplementary file 1 [file jcm-11-02479-s001.zip › jcm-1694235-SI.pdf]
